# BPSD following traumatic brain injury

**DOI:** 10.1590/S1980-57642013DN70300007

**Published:** 2013

**Authors:** Renato Anghinah, Fabio Rios Freire, Fernanda Coelho, Juliana Rhein Lacerda, Magali Taino Schmidt, Vanessa Tomé Gonçalves Calado, Jéssica Natuline Ianof, Sergio Machado, Bruna Velasques, Pedro Ribeiro, Luis Fernando Hindi Basile, Wellingson Silva Paiva, Robson Luis Amorim

**Affiliations:** 1 Center for Cognitive Rehabilitation Post-Traumatic Brain Injury of the Clinicas Hospital of the Division of Neurology, University of São Paulo.; 2 University Salgado de Oliveira, Niterói, RJ and Panic and Respiration Laboratory, (IPUB/UFRJ).; 3 School of Physical Education (EEFD/UFRJ) and National Institute of Traumatology and Orthopaedics (INTO-RJ).; 4 Laboratory of Brain Mapping and Sensory-Motor Integration (IPUB/UFRJ).; 5 Psychophysiology Laboratory, Universidade Metodista de São Paulo & High-Resolution EEG Section.; 6 Division of Neurosurgery, University of São Paulo Medical School.

**Keywords:** TBI, traumatic brain injury, BPSD, treatment

## Abstract

Annually, 700,000 people are hospitalized with brain injury acquired after traumatic brain injury (TBI) in Brazil.

**OBJECTIVE:**

We aim to review the basic concepts related to TBI, and the most common Behavioral and Psychological Symptoms of Dementia (BPSD) findings in moderate and severe TBI survivors. We also discussed our strategies used to manage such patients in the post-acute period.

**METHODS:**

Fifteen TBI outpatients followed at the Center for Cognitive Rehabilitation Post-TBI of the Clinicas Hospital of the University of São Paulo were submitted to a neurological, neuropsychological, speech and occupational therapy evaluation, including the Mini-Mental State Examination. Rehabilitation strategies will then be developed, together with the interdisciplinary team, for each patient individually. Where necessary, the pharmacological approach will be adopted.

**RESULTS:**

Our study will discuss options of pharmacologic treatment choices for cognitive, behavioral, or affective disorders following TBI, providing relevant information related to a structured cognitive rehabilitation service and certainly will offer an alternative for patients and families afflicted by TBI.

**CONCLUSION:**

Traumatic brain injury can cause a variety of potentially disabling psychiatric symptoms and syndromes. Combined behavioral and pharmacological strategies, in the treatment of a set of highly challenging behavioral problems, appears to be essential for good patient recovery.

## Abstract (pt)

Anualmente, 700 mil pessoas são hospitalizadas com lesão encefálica adquirida após traumatismo cranioencefálico (TCE) no Brasil.

**OBJETIVO:**

Nossa meta é revisar os conceitos básicos relacionados ao TCE, e aos Sintomas Comportamentais e Psicológicos da Demência (BPSD) encontrados nos sobreviventes de TCE moderado e grave. Também discutimos as estratégias utilizadas para lidar com os pacientes pós-TCE.

**MÉTODOS:**

Quinze pacientes ambulatoriais acompanhados no Centro de Reabilitação Cognitiva pós-traumatismo cranioencefálico do Hospital das Clínicas de São Paulo foram submetidos a avaliações neurológica, neuropsicológica, fonoaudiológica e de terapia ocupacional, incluindo o mini exame do estado mental. Em seguida, estratégias de reabilitação serão desenvolvidas, com a equipe multidisciplinar, para cada paciente individualmente. E, se necessário, a abordagem farmacológica será adotada.

**RESULTADOS:**

Nosso estudo irá discutir as opções de escolha de tratamento farmacológico para desordens cognitivas, comportamentais e afetivas pós-TCE, fornecendo informações relevantes relacionadas a um serviço de reabilitação cognitiva estruturada e, certamente, irá oferecer uma alternativa para pacientes e famílias vítimas de TCE.

**CONCLUSÃO:**

O traumatismo cranioencefálico pode causar uma variedade de sintomas e síndromes psiquiátricos potencialmente incapacitantes. As estratégias farmacológica e comportamental combinadas para o tratamento de um conjunto de problemas comportamentais muito desafiador parece ser essencial para uma boa recuperação do paciente.

## INTRODUCTION

Brazilian data indicate that about 700,000 people suffer TBI annually, of whom 20 to 30% suffer moderate or severe TBI. The data suggests that 80% of those who suffer mild TBI are able to return to work, whilst only 20% of moderate and 10% of severe TBI cases can return to their daily routine.^1,2^

Traumatic brain injury (TBI) is a nondegenerative, noncongenital insult to the brain from an external mechanical force, potentially leading to permanent or temporary impairment of cognitive, physical, and psychosocial functions, with an associated diminished or altered state of consciousness.^3^

In this context, we will discuss an approach to manage patients with cognitive, behavioral, or affective disorders, rather than define specific pharmacological treatments, in outpatients followed at the Center for Cognitive Rehabilitation Post-Traumatic Brain Injury of Clinicas Hospital of the University of São Paulo, Brazil. The physiological bases for choosing a pharmacological agent to treat such patients, according to the latest research, supports the use of specific drugs for the treatment of specific disorders following TBI.

**Severity levels of TBI.** The level of consciousness and/or coma in the first 24 hours and the time of Post Traumatic Amnesia (PTA) after the TBI are the most used references to differentiate them into mild, moderate or severe injuries. The most widely used method of classifying TBI cases is via a scoring system called the Glasgow Coma Scale (GCS), which assesses the ability of eye opening, motor and verbal responses as determinants for severity evaluation. GCS scores range from 3 to 15, defining that scores less than or equal to 8 indicate severe injury, from 9 to 12, moderate injury, and from 13 to 15, mild TBI.^4^

Post Traumatic Amnesia starts in the period of coma and lasts until the time when the patient recovers their memory consistently. This period can be associated with a transitory state of disorientation, agitation and behavioral disturbances such as insomnia, agitation, confabulation and, occasionally, serious affective and psychotic symptoms.^5^

One of the most used scales to evaluate Post Traumatic Amnesia is the GALVESTON Orientation and Amnesia Test. Typically, in post-TBI patients, personal orientation is recovered before spatial orientation and the perception of what happened. Temporal orientation is the last faculty to be recovered.

TBI severity is attributed at the first moment from the Glasgow scale, coma and Post Traumatic Amnesia period. A TBI is considered severe when the GCS is from 3 to 8, the period of coma is longer than 6 hours and the PTA period is longer than 24 hours; in a moderate TBI the GCS is from 9 to 12, the period of coma shorter than 6 hours and the PTA from 1 to 24 hours; mild cases have a GCS from 13 to 15, a coma lasting for 20 minutes or less, and a PTA of 1 hour or shorter.

Despite the support of the scales, the residual consequences post-TBI will differ in each patient, but the majority who suffer mild trauma have a recovery process without major complications, returning to their pre-trauma activities. On the other hand, the majority of patients who suffer moderate and severe TBI will present sequelae and limitations. However, some patients, despite having suffered only mild trauma, have post trauma repercussions that will require special care and attention from specialized professionals.

**Most common findings in moderate and severe TBI survivors after hospital discharge.** This group of patients is characterized by the diffuse axonal injury and secondary complication to the site or sites of injury.

After the most critical phase of hospital management, most patients return home. Although some patients manage to regain some degree of independence in their self-care, they are still incapable of applying critical thinking to decision-making processes, providing for the needs of their families or continuing work, school or social activities, which can cause difficulties in family relationships and lead to poor quality of life for patients and their relatives. Moreover, these patients may present with mood alteration and depression.

The rehabilitation of these patients after hospital discharge is aimed at a community integration program with day center resources that provide continuity of patient care so that the individual receives vocational and professional training, integrated as part of the rehabilitation process.^6^

One of the scales used in our Centre that systematically seeks to standardize the functional level of post-TBI patients and establish the potential of their rehabilitation management, is the Rancho Los Amigos levels of cognitive functioning scale.^7^ This scale is also widely used in many other centers worldwide. In addition, another scale of diffuse axonal injury rehabilitation stages developed by Tuel^8^ and modified by Katz^9^ is also quite useful at this stage.

**Most common cognitive impairments following TBI.** The type and degree of cognitive impairment following TBI may vary widely, depending on the severity and site of injury. If a focal brain injury occurs, the consequence may be similar to the injury caused by a CVA, such as aphasia, apraxia, unilateral neglect or visuospatial dysfunction. However, these are the typical findings following TBI. Due to the mechanisms of acceleration-deceleration that usually damage the ventral and lateral regions of the frontal and temporal lobes, the most commonly found sequelae are attention and memory deficit, difficulty learning new information, resolving problems, planning, as well as problems associated with impulsivity and self-control. Some "subclinical" findings such as changes in naming, verbal fluency and auditory discrimination are also reported.

Initially, attention deficits are the most common and severe in the residual stage, usually involving difficulty maintaining divided attention.

The long-term memory is generally restored, but some individuals continue having difficulties in learning new things and retaining new information. Working memory is frequently affected including the stages of encoding, storage and retrieval of information. Such changes exert a significant impact on social and vocational reintegration.^10,11^

Some individuals are left with amnesic syndrome, which is more common in those who have gone through periods of hypoxia and anoxia.

Executive functions may be affected, being related to frontal lobe damage. When the frontal injury is severe the patient may be inert or lack initiative (medial or lateral frontal injury), or display inappropriate and impulsive behavior. Many individuals with frontal lobe injury in post-TBI retain much of their skills but are unable to initiate, sequence, organize or monitor their actions so as to meet the targets or goals set.

The language disorders most commonly observed occur in the discursive (tendency to produce irrelevant information and omit important information), pragmatic (loss in production of inferences, difficulty in formulating arguments) and conversation levels (loss of initiative and maintenance of topics with inconsistent switching without signaling). These changes correlate with cognitive impairments in attention, memory and slow mental processing.^6^

The inability to curb impulsive reactions leads to social and family relationship problems. Patients usually have poor self-critical awareness regarding their condition and behavioral changes.

**long-term neuropsychiatric sequelae.** Once patients have survived the acute and subacute phases, many are left with neuropsychiatric impairments. Common issues related to symptoms include behavioral outbursts, emotional adjustments, cognitive deficits, and physical concerns.

The link between ability to adjust to TBI and the capacity to do so is complicated by a major factor. In addition to having to adjust to dysregulation in behavior, emotions, thinking, and physical challenges, persons with TBI must make these adjustments with a brain that processes information poorly. A person with limited coping skills may become frustrated with his or her mood variability or with limb weakness, and may as a result have outbursts. Problems with attention, decreased ability to understand, and difficulty remembering things may impact an individual's ability to adjust to deficits.

The patient may lose the information given at the doctor's office, or may forget to use the assistive device. He or she may have problems perceiving situations or attribute incorrect motives to the actions of others.^12^

**BPSD - most common syndromes post-TBI.** The most common syndromes post-TBI are behavioral disinhibition, depression/anxiety/psychosis, substance abuse and attention/cognitive disorders.

Post-traumatic stress disorder (PTSD) occurs in up to 27% of cases, including patients with no clear recall of the event. Depression has an incidence of 15% to 33% and prevalence of 18% to 42%. Mania occurs in <10% of patients with TBI.^8^ Aggression occurs in variable frequencies (ranging from 20% to 49%) and psychosis occurs in <10% of the TBI population.^12^

**Nnoradrenergic medication.** Noradrenergic agonists are used with varying degrees of success for the behavioral sequelae of brain injury, such as problems with attention, impulsivity and speed of cognitive processing.^13^

Three commonly studied noradrenergic medications are amphetamine, methylphenidate, and L-threo-3,4-dihydroxyphenylserine (L-DOPS). Amphetamine enhances norepinephrine release from nerve terminals, methylphenidate blocks the reuptake of NE, and LDOPS is a precursor of norepinephrine.^14^ A recent Cochrane review concluded that there is insufficient evidence to support the routine use of methylphenidate or other amphetamines.^15^

**Dopaminergic medication.** Most dopaminergic cell bodies reside in the substantia nigra or hypothalamus. Dopa-mine appears to be important in memory, arousal, and executive function. Medications that affect the dopaminergic system are used to treat disorders such as Parkinson disease, attention deficit hyperactivity disorder, and schizophrenia.^16^

McDowell et al. (1998) used bromocriptine in a double-blind, placebo-controlled trial of 24 subjects who had suffered moderate to severe TBI at least 4 weeks before treatment. The patients showed a significant improvement on several tasks thought to specifically represent prefrontal lobe function.^17^

Schneider et al. (1999) found no difference in the rate of cognitive recovery with amantadine treatment in a double-blind, placebo-controlled crossover trial of 10 subjects with moderate to severe TBI.^18^ A retrospective review by Passler et al. (2001) reported that patients given bromocriptine recovered faster from a vegetative state secondary to TBI.^19^

It is believed that dopamine has a role in brain injury rehabilitation, either as a primary effect or through its metabolism into norepinephrine.^14^

**Serotonergic medication.** Serotonin has behavioral effects and is involved in motor control.^20^ The study of Loubinoux et al. (2002) found that a single dose of paroxetine increased functional MRI (fMRI) motor cortex activation in human subjects during a simple motor paradigm.^21^ Another serotonin reuptake inhibitor, fluvoxamine, has been shown to improve reaction time.^22,23^ Serotonergic drugs have not been shown to be effective in reducing symptoms after brain injury in human beings.^14^

In severe TBI, BAY x 3702 was administered in a multicenter, randomized double-blind, placebo-controlled parallel group study. BAY x 3702 was found to be safe, but the effect on outcome was not significant.^24^

Serotonergic medications are often prescribed to patients recovering from TBI because of the relative safety of, and their role in, treating disorders of mood and behavior. Data from animal and human studies suggest that these drugs are safe when given during rehabilitation after traumatic or ischemic brain injury. It is still unclear if serotonergic medication has only immediate effects on behavior or whether they have a long-lasting effect on neuronal recovery.^14^

**Cholinergic system.** Acetylcholine is produced in several nuclei of the brain including the nucleus basalis of Meynert and several tegmental nuclei, with widespread projections to the cerebral cortex, hippocampus, amygdala, hypothalamus, cingulum, and thalamus.^25^ Acetylcholine (Ach) can act as a neuromodulator and change the magnitude of other synaptic events. ACh was the first neurotransmitter targeted to improve recovery after brain injury.^14^

Acetylcholine is involved in memory and recently the use of cholinergic drugs in Alzheimer disease was approved. There is growing evidence of the role of ACh in experience-driven cortical plasticity.^14^ Cerebrospinal fluid levels of ACh fluctuate after TBI, rising in the acute phase followed by a prolonged period of depression.^26,27^ Cholinergic circuits are vulnerable to injury.^14^

Damage to the cholinergic basal forebrain septohippocampal pathways results in severe depletions of ACh and has been associated with learning and memory deficits.^28,29^

In the study of Cardenas et al. (1994) – a blinded, controlled trial of 36 subjects after TBI – a significant number demonstrated improved memory and balance while taking the drug.^30^

Donepezil, an anticholinergic medication used to treat Alzheimer disease and approved by the Food and Drug Administration (FDA), has been reported to improve memory problems after TBI, at least during the period while medication is taken.^31,32^

More research is needed to better define the role of ACh in recovery after TBI.

## METHODS

**Subjects.** This was a retrospective study of fifteen patients with a diagnosis of traumatic brain injury followed at the Outpatient Clinic of the Clinicas Hospital of the University of São Paulo.

**Strategies to manage post-TBI patients.** *Cognitive function evaluation –* The patient will initially be subjected to a reading test, which consists of reading simple children's books allowing evaluation of how long they focus on the book. According to the performance and education of each individual, slightly more complex texts will be presented.

After this first test, each patient will be classified according to the Rancho Los Amigos scale and only those who score greater than or equal to 5 (on a scale of 8) will be referred for cognitive rehabilitation.^33^

This is necessary because, in order to be rehabilitated, the individual must maintain a minimum time set in the task of over 10 minutes.

The next intervention will be to conduct a neuropsychological evaluation, speech and occupational therapy including the Mini-Mental State Examination^34,35^ and clock drawing test. The neuropsychological assessment includes the evaluation of affective/emotional state (BECK scale – BDI, BAI, BHS, BSI),^36^ I.S.S.L^37^ and GDS,^38^ activities of daily living PFEFFER,^39^ batteries of tests of executive functions Wisconsin,^40^ Stroop (Stroop Interference Test – Victoria version) -freely translated from the English version.^41,42^ The Rey complex Figure,^43^ WAIS-III,^44^ attention,^44^ the Wechsler Digit Symbol-Coding, trail making parts A and B (Trail Making Test),^45^ visuo-constructive,^44^ language FAS and Verbal Fluency,^46^ memory,^44^ and Rey Auditory-Verbal Learning Test (RAVLT)^43,47^ will also be applied. All tests have been validated for use in Brazil with scores for different levels of schooling.

The speech evaluation includes pragmatic assessment, according to precepts of conversational analysis, a test of verbal working memory for auditory input (N-Back) language evaluation, the Arizona Battery for Communication Disorders – ABCD.^48^

The tests applied have been validated for use in Brazil with scores according to different levels of schooling except for the "Test of Practical Judgment" (TOP-J), which does not have a version in Portuguese and the Arizona Battery for Communication Disorders – ABCD, which is undergoing the validation process, and will be included in our battery. Computerized tests that evaluate response time for a particular task will also be conducted.

Occupational therapy evaluates cognition in order to determine the individual's capacity to live alone safely and comfortably, to work or undertake any activity they deem important or meaningful^49^. Also, it limits the impact of deficits in memory, attention and executive functions in performing the activities of daily living. The evaluation of performance in BADL and IADL requires the observation of the individual's behavior in the context in which they conduct these activities. The information obtained will be used to develop strategies, with the individual and their family, which will minimize the losses in each cognitive deficit.

Through the evaluation of cognitive abilities in the tasks, it is possible to determine the patient's strengths, limitations and challenges in learning abilities and environmental strategies that will support their daily life.^50,51^ Assessment in Occupational Therapy includes LOTCA^52,53^.

After the neuropsychological, speech and occupational therapy evaluation, rehabilitation strategies will be developed, together with the interdisciplinary team, for each patient individually.

*Pharmacological treatment approach–* Brain injury confers increased sensitivity to the active agents of the central nervous system (CNS); therefore, treatment and side effects may be accentuated at lower doses in patients after TBI.

Secondly, to systematically approach treatment in TBI beyond pharmacological interventions, three areas are important to bear in mind: [1] identification of target symptoms; [2] consideration of coexisting medical problems and iatrogenic contributions; and [3] implementation of nonpharmacological treatment.

The initial steps of treatment include a comprehensive neuropsychiatric evaluation and testing. This may entail neurological and psychiatric examination to document baseline deficits, diagnoses, and functioning.

A neuropsychological battery documents the cognitive skills and limitations, and provides a baseline from which gains in cognitive rehabilitation can be bench-marked. Neurophysiological tests may show brain dysfunction and seizures. When indicated, neuroimaging may be of benefit to show ischemia, hemorrhage, encephalomalacia, neuronal loss, and altered cerebral metabolism or perfusion.^7^

The pharmacological approach focuses on functional recovery and its target of therapy is symptom reduction through pharmacological enhancement of rehabilitation. Most drugs affect more than one neurotransmitter systems, in various parts of the nervous system. After a TBI, neurotransmitter levels change and may be a target of therapy. Glutamate and aspartate are released excessively, cortical levels of dopamine are suppressed for at least several weeks after TBI. The levels of norepinephrine and acetylcholine fluctuate, with an initial increase followed by prolonged decrease.^9^

After the evaluations, rehabilitation strategies will be developed, in conjunction with the interdisciplinary team, for each patient individually. And if necessary, the pharmacological approach will be adopted.

## RESULTS

Our experience using the Pharmacological TBI Guideline approach for 15 outpatients with BPSD showed that the patients improved with the use of mood stabilizers, selective serotonin reuptake inhibitors and dopaminestimulating drugs. The causes of TBI and the drugs in use among the TBI patients are shown in Table 1.

**Table 1:** Profile of TBI patients.

Patient	Gender	Causes of TBI	Medication in use
1	M	Run over by a car	Valproic acid, venlafaxine
2	M	Fall	Valproic acid, amantadine, venlafaxine
3	F	Motorcycle accident	Venlafaxine
4	F	Car accident	Valproic acid, modafinil, donepezil
5	M	Fall	Quetiapine
6	M	Car accident	Venlafaxine
7	F	Run over	Valproic acid
8	M	Fall	Promet, amitriptyline, donepezil
9	M	Fall	Haloperidol, bipyridine, aglometadine
10	M	Physical aggression	Amitriptyline, alprazolam, buspirone
11	F	Run over by a bus	Valproic acid
12	M	Fall	Quetiapine
13	M	Physical aggression	Venlafaxine
14	F	Run over by a motorcycle	Neuleptil, venlafaxine
15	F	Fall (hot-air balloon)	Olanzapine, venlafaxine, amitriptyline

## DISCUSSION

Valproic acid – a mood stabilizer – and venlafaxine – a drug that has dual action with selective serotonin reuptake inhibitors (SSRI) and adrenergic reuptake, in addition to weak action as a dopamine stimulant – are the two medications with the best indication for TBI in our milieu, where possible replacing with hidantal (phenytoin) or gardenal (phenobarbital) when the patient uses neuroleptics. An anticholinesterase inhibitor, originally developed for Alzheimer's disease, is used for improving memory.

Amantadine – a weak antagonist of the NMDA type glutamate receptor, that increases dopamine release and blocks dopamine reuptake – is a good option for enhancing attention. It has been used for Parkinson's Disease. Modafinil was used to treat hypersomnia.

**Motivation.** Lack of motivation is very common after a significant head injury. Injuries to the orbitofrontal, medial frontal cortical, ventral pallidum and ventral tegmentum may all affect motivation.^54^ A poverty of behavior and speech, lack of initiative, loss of emotional responses, and severe psychomotor problems can be classified as a more extreme form of abulia. Both pharmacological treatment and behavioral interventions are necessary to treat these syndromes. Stimulants and dopaminergic medications, as well as activating antidepressants such as bupropion or monoamine oxidase inhibitors (MAOIs) are good options. Selective serotonin reuptake inhibitors (SSRIs) and typical neuroleptics are bad choices, worsening these syndromes.^55^

**Mood disorders.** Following TBI, up to 50% of patients have symptoms of depression, with 20% meeting Diagnostic and Statistical Manual of Mental Disorders, Fourth Edition (DSM-IV) criteria for major depression. These patients have a significantly increased risk of suicide.^12^

Tricyclic antidepressants (TCAs) may be a good option to treat anxiety and depression^56^ but they should be limited to agents with the least anticholinergic activity, the least sedation, and the least effect on the seizure threshold. Low histamine, cholinergic, and *a*-adrenergic binding SSRIs are preferred for mood and anxiety, but side effects of sexual dysfunction, dyspepsia, drowsiness, and irritability may limit their use in TBI. A few studies have shown that stimulants, amantadine, and anticholinesterase drugs are helpful.^57-59^ The drugs of choice for post-traumatic mania are clonidine, carbamazepine, and divalproex. Lithium is reserved for patients with a prior history of mania.^60^

**Psychotic disorders.** The onset of psychotic symptoms is usually delayed following the injury, by around 4 years.^61^ The psychoses are characterized by hallucinations and delusions with retained insight.^12^

TBI psychosis differs from schizophrenia because of the absence of Schneiderian first-rank symptoms in non-delirium associated hallucinations that can occur post-TBI. A later age of onset, less premorbid psychiatric disturbance, briefer duration, less common family history, better response to neuroleptics, less need for maintenance medication, and a better prognosis are other factors distinguishing TBI psychosis from schizophrenia.^62^

Neuroleptics may cause adverse reactions, so it is important to initiate the treatment for post-TBI psychosis at one third to one half the usual doses.^63^ Neuroleptics may impede cognitive recovery.^64^ Atypical neuroleptics, except for clozapine (which is an anticholinergic and lowers the seizure threshold) are the drugs of choice. Benzodiazepines should be used sparingly.^12^

**Post-traumatic stress disorder and other anxiety disorders.** Anxiety can occur in patients who have suffered a TBI, presenting as insomnia, inability to concentrate, free-floating phobias, and panic attacks. Situations in which the patients know that they cannot perform as well as they did before their TBI cause anxiety. It is important that the patient understands the injury and learns how to deal with his/her limitations. In an acute stress situation, a short course of a short-acting benzodiazepine may help. Selective serotonin reuptake inhibitors (SS-RIs) may be helpful but they should be used with care because they can exacerbate apathy and cause sexual dysfunction. Antiepileptic drugs (AEDs) may also be useful, particularly in patients who are aggressive.^12^

**Somatoform disorders.** Somatization may manifest as dizziness, vertigo, gait instability, headache, fatigue, malaise, tinnitus, visual disturbances, cognitive "fogginess," nonneuroanatomical paresthesias, weakness, focal deficits, and nonepileptic events.^12^ Patients presenting these symptoms are often classified as having "postconcussional syndrome".^65^ Cognitive behavior therapy (CBT) may be useful for non-TBI populations but treatment for these disorders are lacking.^12^

**Cognitive disorders.** These disorders are among the most commonly occurring sequelae in TBI and post-traumatic cholinergic deficits are thought to contribute to the development of post-traumatic cognitive impairments.^12^ The study of Silver et al. (2006) – a randomized double-blind placebo-controlled study of a cholinesterase inhibitor in 157 post-TBI patients – showed no difference from placebo in both primary cognitive and secondary outcome measures.^67^

**Personality change due to medical condition.** The personality changes are most often exaggerations of premorbid personality traits and may include lability, disinhibition, aggression, apathy, and paranoia. Impulsivity, lack of empathy, loss of a sense of self, and inability to monitor one's own behavior are typical of the "pseudoborderline" personality disorder. Damage to the orbitofrontal cortex may cause mania, euphoria, and impulsivity that are labeled as "pseudosociopathic syndrome". A "pseudodepressed" personality disorder can be the result of medial frontal damage and may cause severe apathy. It is very common to find explosive personality disorders, particularly among patients who use alcohol. Counseling is necessary for treatment of these various personality changes. The use of tricyclic and SSRI antidepressants may help with lability. Some studies have demonstrated that impulsivity can be controlled with low-dose stimulants, L-dopa, and dopamine agonists.^67^

**Aggressive disorders.** In the delirious subacute phase of recovery, agitation occurs frequently. Aggression in patients with TBI is generally reactive without premeditation, and is nonpurposeful, explosive, periodic, and egodystonic.^68^..

The aggressive behavior is associated with damage to the limbic system, orbitofrontal cortex, left anteromedial frontal lobe, and anterior cingulate.^69^

Many of the agents used for agitation are also used for aggression. Antipsychotic medications do not help with chronic, nonpsychotic aggression. Akathisia is a common adverse reaction to typical neuroleptics and may increase violent behavior. It has been demonstrated that neuroleptics are responsible for slow recovery from TBI.^12^

Benzodiazepines and low-dose buspirone may cause a paradoxical reaction. Antipsychotic agents should be used for patients who are indeed psychotic. The use of carbamazepine, valproic acid, gabapentin and oxcarbazepine has proven successful in some patients. Lithium is a good option in patients who are manic or that display cyclic violence. High-dose propranolol has proven very helpful in some patients. Antidepressant medications, such as TCAs and SSRIs, have been shown to be helpful in a few studies.^70^

In conclusion, traumatic brain injury can cause a variety of potentially disabling psychiatric symptoms and syndromes. These include mood and anxiety disorders; personality disturbances; aggression; and, often, psychosis. The diagnosis of cognitive and behavioral disorders following TBI can be complex. It is therefore essential that the physician obtain a broad picture of the patient before prescribing medications. A differential diagnostic approach can help the physician to sort through the numerous etiological factors that may be contributing to the patient's condition. Pharmacological treatment may include a wide range of medications, such as antidepressants, antipsychotics, mood stabilizers, and stimulants. Family and individual counseling is particularly important in helping the patient and the family reconcile themselves to the reality of the behavioral changes in the patient post-TBI.
